# Development, predictors, and effects of trainees’ organizational identification during their first year of vocational education and training

**DOI:** 10.3389/fpsyg.2023.1148251

**Published:** 2023-04-17

**Authors:** Elisabeth Maué, Stefanie Findeisen, Stephan Schumann

**Affiliations:** ^1^Department of Economics, University of Konstanz, Konstanz, Germany; ^2^Cluster of Excellence “The Politics of Inequality”, University of Konstanz, Konstanz, Germany

**Keywords:** organizational identification, support, formal socialization, social integration, dropout intention, performance, engagement, vocational education and training

## Abstract

The vocational identity of trainees is one component of their professional competence and is considered to be a central goal of vocational education and training (VET) programs. From the numerous identity constructs and conceptualizations, this study focuses on the organizational identification of trainees, that is, the extent to which trainees internalize the values and goals of their training company and perceive themselves as part of this company. We are specifically interested in the development, predictors, and effects of trainees’ organizational identification, as well as the interrelations between organizational identification and social integration. We use longitudinal data of *n* = 250 trainees in dual VET programs in Germany at the very beginning of their VET program (t1), after 3 months (t2), and after 9 months (t3). A structural equation model was used to analyze the development, predictors, and effects of organizational identification for the first 9 months of training and the cross-lagged effects between organizational identification and social integration. The results showed a high stability of trainees’ organizational identification over the first 9 months. Regarding the predictors, the results indicated positive direct and indirect effects of the formal socialization tactics implemented by the training company, as well as of support by the trainer at the beginning of the training. However, collegial support at the beginning of the training did not seem to play a significant role in organizational identification. Moreover, organizational identification positively affected trainees’ emotional engagement and self-perceived competence while negatively predicting dropout intentions after 9 months of training. Finally, the cross-lagged effects between organizational identification and social integration were not significant, and only at t3 were these constructs positively correlated. However, regarding the development, predictors, and effects, very similar results were found for organizational identification and social integration. The results underline the positive significance of organizational identification for the individual, the company, and society, even at this early stage of training. The results are discussed regarding both their scientific and practical implications.

## 1. Introduction

The development of vocational identity is regarded as a central goal of vocational education and training (VET) programs and identity is considered a component of trainees’ professional competence (e.g., Rauner, 2007; Baethge et al., 2009; Heinrichs et al., 2022). Understanding and supporting trainees’ identification processes are especially important aspects in the context of VET because the transition from school to VET is a crucial step for adolescents, determining individuals’ future career success and life (Savickas, 1999; Seeber et al., 2019). When entering a training program, adolescents—for the first time and at a relatively young age—are faced with work adjustment processes, during which they try to achieve (and maintain) congruence with their work environment (Dawis and Lofquist, 1984). The development of vocational identity is a part of this process.

When conceptualizing vocational identity, two foci can be distinguished (for a detailed distinction between the different aspects of the construct identity, see Heinrichs et al., 2022). First, in the more narrow sense of the term, vocational identity is regarded as personal identity construction which is premised on a self-reflective process of individuals (e.g., Frey and Haußer, 1987). This conceptualization is based on conceptions of identity in developmental psychology (Erikson, 1959; Marcia, 1980) and perceives vocational identity as that part of a person’s overall identity that relates to the context of work and occupation. Second, vocational identity can be regarded as a social identity emphasizing an individual’s affiliation with a certain group in the workplace (e.g., Raeder and Grote, 2007). This aspect of the identity construct is typically referred to as identification and is closely linked to Social Identity Theory (Tajfel and Turner, 1979). Social Identity Theory claims that individuals at least partly define themselves *via* their belonging to a certain group, for example, their workplace. Accordingly, while at work, individuals act as members of a group (team, organization, etc.), and their actions are not merely based on their own aims and values but rather on those of the group (Tajfel and Turner, 1979; van Dick, 2017). An individual’s identification mirrors the extent to which they have adapted the group’s values and goals (Mael and Tetrick, 1992).

In general, identification refers to the different aspects of an individual’s professional life. Typical reference points are an individual’s occupation, their team or their organization (van Dick et al., 2004). The present paper focuses on organizational identification, which can be defined as “the degree to which a member defines him- or herself by the same attributes that he or she believes define the organization” (Dutton et al., 1994, 239). Organizational identification is one form of social identity because it refers to an individual’s psychological attachment to a certain organization (Mael and Ashforth, 1992, 1995); individuals internalize the goals and values of their organization and perceive themselves as an integral part of the organization, which means that an organization’s failures and successes are regarded as one’s own failures and successes.

Vocational identity and organizational identification have been shown to be positively correlated (*r* = 0.57–0.74; Klotz et al., 2014; Kirchknopf and Kögler, 2022; also Kirchknopf, 2020). Both constructs are expected to be highly relevant for individuals’ performance of work tasks (e.g., Heinrichs et al., 2022). Evidence from the VET context also shows that, besides performance, identification is related to trainees’ well-being and health, as well as to the organizations’ working climate and customer satisfaction (Thole, 2021). Hence, trainees’ identification is an important factor in the training process. Consequently, organizations have a strong interest in supporting trainees’ work adjustment processes and fostering trainees’ identification as the trainees are starting a VET program—particularly against the backdrop of the shortage of skilled workers and the emerging shortage of young skilled workers (e.g., Dettmann et al., 2019). Since VET programs face rather high numbers of premature contract terminations (e.g., Germany: 27%; Bundesinstitut für Berufsbildung (BIBB), 2022), especially in the probationary period and within the first year, understanding identification processes more thoroughly might be a means to address these problems.

Despite the high relevance of identification in VET programs, Heinrichs et al. (2022) claim that there is still a general need for empirical studies on the identification development of trainees in VET. There is a lack of longitudinal studies examining the development of identification over the course of the training program, as well as insights into beneficial factors that foster the development of identification during VET. At the same time, empirical evidence on the effects of organizational identification on a successful course of the training program (e.g., trainees’ persistence, performance) is also scarce. Our longitudinal study addresses these research gaps and examines (1) the development of trainees’ organizational identification over the first 9 months of VET. Moreover, we are interested in (2) organizational predictors and (3) the effects of trainees’ organizational identification. Finally, we analyze (4) the relationship between trainees’ organizational identification and social integration into the organization.

## 2. Development, predictors, and effects of organizational identification

The core element of vocational and educational training in Germany is the so-called *dual system*. The dual system is a major path-way into skilled employment and also a crucial element of workforce development for many enterprises. Vocational training in the dual system takes place at two “learning venues” (Deissinger, 2010; Greinert, 1994): the training company that offers and funds the training program and the part-time vocational school (*Berufsschule*) where the trainees receive theoretical instruction. There are currently 324 different vocational training programs in Germany (Bundesinstitut für Berufsbildung (BIBB), 2021), lasting between 2 and 4 years.

At the start of dual VET programs, trainees are faced with socialization processes. Socialization processes are the learning processes during which trainees gradually become members of their training organization and their working group (Lave and Wenger, 1991; Ostroff and Kozlowski, 1992; Bauer and Erdogan, 2011). During socialization processes, individuals (1) gain knowledge and skills regarding the performance of their working tasks and (2) integrate into their working group (Morrison, 1993). Trainees’ social integration and their organizational identification/organizational commitment^1^ can be regarded as the outcomes of trainees’ socialization processes (e.g., Reichers, 1987; Morrison, 2002; Nägele and Neuenschwander, 2014). Socialization is guided both by the newcomer’s initiative and the company’s initiative (Morrison, 1993). In the context of VET, support by trainers and colleagues is an important factor in helping new trainees during their socialization processes. Hence, the training organization can implement those measures expected to help trainees’ socialization, for instance, through the use of onboarding programs (Saks and Ashforth, 1997).

The number of studies focusing specifically on organizational identification in the VET context is scarce. In the following, we have, therefore, considered studies examining other identity or identification constructs for the VET context. For other strands of the literature and constructs for which the findings are more detailed, only those studies that examine the construct of organizational identification are included.

### 2.1. Development of organizational identification

For trainees in VET, several studies have examined the development of vocational identity in general, as well as the development of organizational identification across a VET program. For instance, when it comes to vocational identity, Heinemann et al. (2009) show that the development over the course of the 3-year training program is different for different occupations. In most cases, however, vocational identity declines from the first to second year of training. Some cases also show a U-shaped development (higher values in year 3 than in year 2). Fischer (2013) shows that trainees in the field of health care and nursing report high values of vocational identity at the start of the VET program. In contrast to the results reported above, there is no decline over the course of the training program: vocational identity is stable over further measurement points at the middle and end of VET. Moreover, individuals’ vocational identity increases 1–3 years after the VET program ends. Valuable insights into the identification process in VET programs are also provided in a recent study by Kirchknopf and Kögler (2022) regarding trainees in the commercial sector; the longitudinal study shows that organizational identification declines over the course of the VET program. In line with the results of Heinemann et al. (2009), the study reveals significant differences regarding organizational identification reported at the start of the training program between trainees from different occupations; however, with regard to the development of organizational identification, no significant differences are identified between different training occupations.

### 2.2. Predictors of organizational identification

Regarding the predictors of individuals’ identification, existing evidence supports the assumption outlined above that a company’s efforts can support socialization processes and, in turn, individuals’ organizational identification. In the VET context, training conditions seem to play an important role. For instance, Heinemann et al. (2009) show that support by colleagues positively affects trainees’ vocational identity. Furthermore, according to Böhn and Deutscher (2021), organizational identification correlates highly with various facets of support from colleagues and trainers, for example social involvement, professional instruction, and mentoring. In line with this, Klotz et al. (2014) also find that the “functional integration of apprentices into operating working processes” (p. 6) significantly positively predicts organizational identification. Similar results are found by Metzlaff (2015), who shows that transparency regarding the purpose of the trainees’ activities fosters organizational identification. Furthermore, the perceived professional expertise of the trainer, the relationship of the trainees with the trainer, and the working climate are positively related to trainees’ organizational identification (Metzlaff, 2015). Additionally, Forster-Heinzer (2020) demonstrates a positive effect of trainees’ perception of their trainers’ pedagogical ethos that means their caring behavior, fairness, and their presupposition about which tasks trainees are capable of completing, on trainees’ organizational identification. Similarly, findings on employees show that organizational identification is positively correlated with both supervisor support and organizational support (van Knippenberg et al., 2007). Furthermore, the organizational identification of newcomers is affected by an organization’s onboarding efforts (Narayansany and Mat Isa, 2021).

### 2.3. Effects of organizational identification

Trainees’ identification is generally expected to affect different aspects of the trainees’ work in the training company (e.g., Heinrichs et al., 2022). First, Thole (2021) shows that trainees’ identification is positively related to their performance. According to Metzlaff (2015), organizational identification is higher when trainees perceive their tasks as being complex. The results of Klotz et al. (2014) also indicate a significantly positive correlation between organizational identity and both trainees’ self-perceived competence (*r* = 0.16) and their scores on a competence test (*r* = 0.14). Böhn and Deutscher (2021) report an even higher correlation between organizational identification and trainees’ self-assessed professional competence (*r* = 0.48). In a mandatory training program for managers, Kim et al. (2015) find that training performance (behavioral performance assessed by observers) is significantly predicted by participants’ organizational identification. In addition, organizational identification has been shown to be significantly positively related to employees’ continuous efforts to improve the quality of their work processes (Lee, 2004).

Second, the literature has shown a significantly positive correlation between trainees’ vocational identity and their engagement (Heinemann et al., 2009; Fischer, 2013). For trainees in the field of health care and nursing, Fischer (2013) distinguishes between organizational engagement and occupational engagement, showing that both constructs correlate significantly positively with vocational identity (organizational engagement: *r* = 0.43; occupational engagement: *r* = 0.58).

Third, identification has been shown to be related to employees’ turnover intention. A recent study reveals that organizational identification is related to lower turnover intentions by newcomers, acting as a mediator between onboarding efforts and turnover intention (Narayansany and Mat Isa, 2021). Similarly, van Knippenberg et al. (2007) show that there is an interaction effect of organizational identification and support from the organization and colleagues on individuals’ withdrawal from the job (turnover intention and absenteeism). Furthermore, evidence from the turnover literature indicates a significant negative effect of organizational commitment on turnover intention. However, this effect is partially mediated by work engagement. The effect becomes weaker when also accounting for person–supervisor fit as a moderator (Zhang et al., 2015). This underscores the importance that support and social integration have on organizational identification and its effects.

In the context of VET, dropouts/dropout intentions have generally been shown to be related to person–vocation fit (e.g., Nägele and Neuenschwander, 2015; Bosset et al., 2022; Findeisen et al., 2022), as well as training quality (Negrini et al., 2016; Böhn and Deutscher, 2021; Krötz and Deutscher, 2021) and support by trainers and colleagues (Stalder, 2012; Bosset et al., 2022). However, evidence of the relationship between trainees’ organizational identification and dropout (intention) is still rather scarce. The results of Kirchknopf and Kögler (2022) suggest that the effects are comparable to those reported in the turnover research; their study reveals that the higher trainees’ initial identification, the lower their intention to change the training occupation or training company. Furthermore, a higher amount of decline in organizational identification leads to a higher intention to terminate the training contract prematurely.

### 2.4. Organizational identification and social integration

Because both organizational identification and social integration are supposed to be fostered during individuals’ socialization processes (see above), the two constructs are assumed to be interrelated. During the socialization process, integration into a group of colleagues can help establish an identity with the new organization (Reichers, 1987). Accordingly, Kammeyer-Mueller and Wanberg (2003) refer to work group integration as the proximal outcome of newcomers’ adjustment and organizational commitment as the distal outcome of adjustment. However, empirical evidence on this relationship is still scarce; to the best of our knowledge, very few studies have examined the relationship between these two constructs. In the context of VET, Metzlaff (2015) finds a positive effect of social integration on organizational identification. The results of Kirchknopf and Kögler (2022) indicate that if the training conditions meet individuals’ basic needs in terms of Self-Determination Theory (Deci and Ryan, 1993; e.g., social integration), the negative development of identification can partly be compensated. Nägele and Neuenschwander (2014) show that trainees’ organizational commitment is significantly predicted by their social integration. Kammeyer-Mueller and Wanberg (2003) obtain comparable results for newcomers.

The assumption that organizational identification and social integration are positively related is further underlined by the similarities regarding (1) their development during VET, (2) their predictors, and (3) their effects. First, as for organizational identification, trainees report high values of social integration at the beginning of VET, and their assessment declines over the first few months into the training program (Nägele and Neuenschwander, 2016; similar results are reported for newcomers in the meta-analysis of Bauer et al., 2007). Second, company initiatives and support also predict individuals’ social integration. In a recent study, Findeisen et al. (2022) show that social integration during VET programs is significantly positively related to the relationship with the trainer. For newcomers, Kammeyer-Mueller and Wanberg (2003) find that efforts by the organization, leaders, and work group positively affect individuals’ work group integration. Third, social integration has been shown to affect turnover (intention; for trainees in VET, e.g., Findeisen et al., 2022; for newcomers, e.g., Bauer et al., 2007) and performance (for newcomers, e.g., Bauer et al., 2007).

## 3. Present study

As outlined above, from the start of their training, organizations have a strong interest in fostering trainees’ identification with the company. Particularly against the backdrop of premature contract terminations in VET, it is highly relevant to investigate the development, predictors, and the effects of organizational identification so that the companies can effectively support trainees in their socialization processes and their development of organizational identification.

Our study focuses on the first year of training because the initial phase of VET has been shown to be the most critical regarding the school-to-work transition (Stalder, 2012), and this is also the period that is most commonly related to premature contract terminations (Frey et al., 2014; Bundesinstitut für Berufsbildung (BIBB), 2022). We have used longitudinal data that contain information on German trainees during their first year of VET. The data set includes three different measurement points: t1: beginning of the VET program, t2: 3 months into the VET program, and t3: 9 months into the VET program.

We examine the following research questions. First, we are interested in the development of trainees’ perceived organizational identification over the course of their first nine months in VET.

1. How does organizational identification develop over the first 9 months of vocational training programs?

Based on the results reported above (see Heinemann et al., 2009; Kirchknopf and Kögler, 2022), we expect organizational identification to decline over the course of the first year of training (Hypothesis 1).

In addition, we aim to identify the predictors of organizational identification. Hence, we focus on the measures applied by the training company during the initial phase of a VET program.

1. How does the support provided by the training company affect organizational identification?

**Figure 1:**
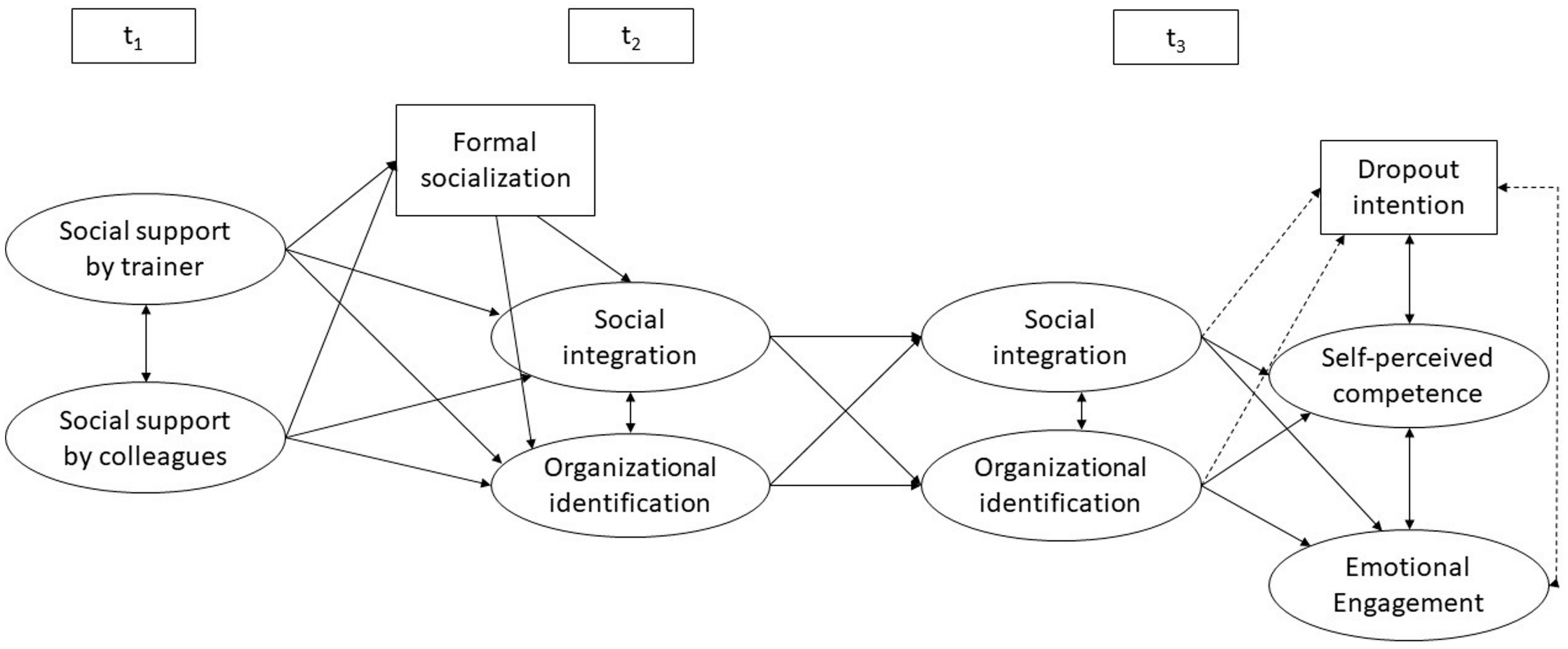
Hypothesized relationships between different predictors and outcomes of organizational identification. Solid lines: positive correlations/effects; dashed lines: negative correlations/effects.

We hypothesize the relationships that are depicted in Figure 1. We expect organizational identification to be positively affected by both social support received by the trainer (Hypothesis 2; see van Knippenberg et al., 2007; Metzlaff, 2015; Forster-Heinzer, 2020) and colleagues (Hypothesis 3; see Heinemann et al., 2009) at the beginning of the training period and by formal socialization tactics employed by the company at the start of the VET program (Hypothesis 4; see Klotz et al., 2014; Narayansany and Mat Isa, 2021).

Furthermore, we are interested in the effect of organizational identification on trainees’ self-perceived competence, their emotional engagement with respect to their work tasks, and their dropout intention.

1. How does organizational identification affect (a) self-perceived competence, (b) emotional engagement, and (c) dropout intention?

In line with prior research (e.g., Lee, 2004; Heinemann et al., 2009; Klotz et al., 2014; Kim et al., 2015; Thole, 2021), we expect organizational identification to be a positive predictor of performance (Hypothesis 5) and emotional engagement (Hypothesis 6). We use self-perceived competence as an indicator of trainees’ performance and, to better differentiate from performance, the emotional component of engagement as an indicator of engagement. Furthermore, organizational identification can be seen as a protective factor for dropping out of training (Narayansany and Mat Isa, 2021; Kirchknopf and Kögler, 2022), so we expect a negative effect of organizational identification on dropout intention (Hypothesis 7).

Finally, we are especially interested in the relationship between trainees’ organizational identification and their social integration.

1. How are organizational identification and social integration interrelated?

As described in Section 2, both constructs can be seen as important outcomes of trainees’ socialization processes at the beginning of their VET program. Hence, a certain relationship between the two constructs can be expected, especially because both constructs seem to be equally supported by similar initiatives by the company and produce similar effects (see Section 2.4). For this reason, we additionally model the development, predictors, and effects of social integration analogous to organizational identification (see Figure 1), even though they are not the main focus of the study. The evidence points toward a positive effect of social integration on organizational identification (Metzlaff, 2015; Kirchknopf and Kögler, 2022) or organizational commitment (Nägele and Neuenschwander, 2014). However, there is still a need for a more thorough analysis of whether and how the constructs significantly predict each other. To examine this relationship more closely, we model and analyze cross-lagged effects (see Figure 1).

## 4. Materials and methods

### 4.1. Data collection and sample

The present paper was prepared within the framework of the ongoing interdisciplinary research project *integration@work*^2^ in the fields of economic education, politics and public administration, and sociology. The aim was to understand under which conditions the integration of trainees in companies is successful, that is, their vocational training is successfully completed.

With the support of the Association of German Chambers of Industry and Commerce (DIHK) and the German Confederation of Skilled Crafts (ZDH), companies from various sectors throughout Germany that train adolescents were recruited. The trainees who agreed to participate in the project were registered for the study by their respective trainers. They received a QR code and participated anonymously in subsequent surveys *via* a smartphone app. To enable all trainees to participate in the surveys, the questionnaires were translated into 10 languages in addition to the German version (Albanian, Arabic, English, Farsi, French, Polish, Russian, Serbian, Tigrinya, and Turkish). For participating in the surveys of the study, trainees received monetary incentives.

**Figure 2:**
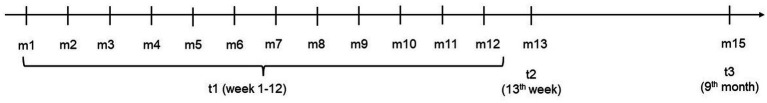
Overview of the different survey times.

The longitudinal study contained several measurement points (see Figure 2). At the start of the in-company training, trainees participated in 12 short surveys at weekly intervals (about 5–10 min each), for example, assessing the perceived quality of the relationship with the trainer or the colleagues. This sequence of measurements is referred to as t1 in our analysis. One week after the last weekly survey, the trainees received the first of a total of 11 more extensive questionnaires (about 10 min each), which were carried out at intervals of 3 months. Here, the trainees, for instance, reported organizational identification. In our analyses, out of the subsequent measurements, we have used the measurement after 13 weeks into the training program (t2; which also marks the end of the typical probationary period in dual VET programs) and the measurement after 9 months (t3).

The sample for the analyses included trainees who started their training program in August or September 2021 (with a few exceptions). We selected those trainees who participated in at least six out of the first 12 measurements of t1 and at t2 and/or at t3 (*n* = 250).

The gender ratio was roughly balanced, with 44% young women and 56% young men. On average, the trainees began their training at age 21 (*M* = 21.19, *SD* = 4.48), with the age range being from 16 to 44 years. More than half (54%) of the trainees were natives (adolescents and parents born in Germany), 21% were born in Germany, but their parents were not (second generation), and 25% had immigrated themselves (first generation). Two-thirds (67%) of the adolescents stated German as their mother tongue and one-third (32%) another language. Out of all the trainees, 9% had no school-leaving qualifications, 7% had a lower secondary school-leaving certificate, 38% had an intermediate school-leaving certificate, and 46% acquired a higher education entrance qualification.^3^ The various training occupations can be assigned to three major occupational fields, with commercial training accounting for the largest share of 45%, followed by 39% in the craft trades. The remaining 16% attended a training program in the field of care (nursing/medicine/education). Most young people (94%) completed their training in companies with more than 250 employees.

Because the companies or trainers selected the trainees to participate in the study, a fundamental bias within the sample in terms of motivation or the like was rather unlikely. However, the extent to which certain characteristics could influence consistent participation in the surveys and persistence in the training would have to be investigated in further analyses. For the constructs included in this study, dropout analyses showed no selection bias: the trainees included in the analyses did not differ significantly from those excluded for the analyses in terms of gender, age, migration background, highest school-leaving qualification, and support received by the trainer and the colleagues at the beginning of their VET program (see Table A1 in the Supplementary Material).

### 4.2. Instruments

Table 1 shows the instruments used. Except for the assessment of dropout intention, we relied on established scales. The original items were translated and slightly modified in terms of language for the target group of trainees. Table 1 reports all constructs and respective measurement points, reliabilities of the scales, and example items. Please note that formal socialization was assessed at t2; however, the trainees retrospectively evaluated formal socialization at the beginning of their training program.

**Table 1:** Overview of the instruments used.

Construct (timepoint)	Number of items	Cronbach’s *α*	Example item
Social support by trainer (t1)	3	0.84	How did the person who gave you most of the tasks this week behave? That person… …helped me (Vinokur et al., 1996, 169).
Social support by colleagues (t1)	3	0.85	How did your colleagues behave this week? The colleagues…helped me (Vinokur et al., 1996, 169).
Formal socialization (t2)	Single item		I have been introduced to my apprenticeship in such a way that I have quickly acquired important knowledge and skills (Jones, 1986, 278).
Social integration (t2, t3)	4	0.89 (t2)	I feel comfortable with my colleagues (Morrison, 1993, 2002, 175, 1153).
		0.88 (t3)	
Organizational identification (t2, t3)	4	0.75 (t2)	The successes of this company are also my successes (Mael and Ashforth, 1992, 122).
		0.82 (t3)	
Self-perceived competence (t3)	3	0.65	I feel competent in the execution of my tasks (Morrison, 1993, 2002, 175, 1154).
Emotional engagement (t3)	3	0.90	My work inspires me (Rich et al., 2010, 634).
Dropout intention	Single item		In the past 3 months, how often have you seriously considered to discontinue your apprenticeship?

For all the items, the response scale was a five-point Likert scale (1 = not true, 2 = rather not true, 3 = some, some, 4 = rather true, and 5 = true). Dropout intention was evaluated on a slightly different scale (1 = never, 2 = once at most, 3 = from time to time, 4 = quite often, and 5 = very often/always). With the exception of self-perceived competence, the reliability of the scales used was found to be at an acceptable to good level (Field, 2009).

An overview of the correlations between the constructs used can be found in Table 2. All but one of the correlations exceeded the 0.10 threshold for low correlations (Cohen, 1988), so overall, we found small, medium, and high correlations, all but three of which were significant. In particular, the autocorrelations of social integration and organizational identification between t2 and t3 turned out to be high. Moreover, there was a strong positive correlation between the two measures of social support.

**Table 2:** Correlations between the constructs.

	Construct timepoint	1	2	3	4	5	6	7	8	9	10
1.	Social support by trainer (t1)	–	**0.60**	**0.38**	**0.43**	**0.43**	**0.31**	**0.37**	0.08	**0.43**	**−0.18**
2.	Social support by colleagues (t1)			**0.39**	**0.57**	**0.55**	**0.26**	**0.34**	**0.20**	**0.45**	**−0.27**
3.	Formal socialization (t2)				**0.39**	**0.40**	**0.30**	**0.32**	**0.22**	**0.33**	**−0.27**
4.	Social integration (t2)					**0.57**	**0.29**	0.18	0.13	**0.25**	**−0.25**
5.	Social integration (t3)						**0.26**	**0.40**	**0.49**	**0.57**	**−0.42**
6.	Organizational identification (t2)							**0.64**	**0.25**	**0.42**	**−0.22**
7.	Organizational identification (t3)								**0.40**	**0.58**	**−0.40**
8.	Self-perceived competence (t3)									**0.45**	**−0.30**
9.	Emotional engagement (t3)										**−0.49**
10.	Dropout intention (t3)										–

Correlations which are significant at the 5% level are in bold.

### 4.3. Data analysis

To keep the survey effort for the trainees low and the sample as large as possible, the items on support from trainers and colleagues during the first weeks of training were asked in weekly alternation. In the six surveys within odd training weeks (m1, m3,…m11), support by the trainer was reported; in the six surveys within even training weeks (m2, m4,…m12), trainees indicated the support received by colleagues. To obtain an overall impression of the support provided by the trainer and colleagues during the first weeks of training, the first step in the analysis was to calculate the mean value per item across the total of six surveys on support from the trainer and from colleagues. In the second step, the two scales for support were modeled from the three items averaged over the six surveys.

In addition to descriptive statistics and correlations, potential changes between 3 months into the training program (t2) and after 9 months of training (t3) were examined using paired-sample *t*-tests. Effect sizes are expressed as Cohen’s *d*, where *d* ≥ 0.2 indicates a weak effect, *d* ≥ 0.5 indicates a medium effect, and *d* ≥ 0.8 indicates a strong effect (Cohen, 1988). These analyses were performed using SPSS version 29.

Structural equation modeling was conducted with Mplus version 8.8 (Muthén and Muthén, 1998–2017). To account for missing data, we used the full information maximum likelihood (FIML) procedure (Jia and Wu, 2019). Furthermore, the maximum likelihood robust (MLR) estimator was used to account for non-normally distributed data. We used 20 random starts to replicate the best-fit function several times. In the structural equation model, we have specified the paths depicted in Figure 1. First, we checked the factor loadings, which were significant for all items (*p* < 0.001) and, with only one exception, substantial in size (≥ 0.50). Further information can be found in Table A2 in the Supplementary Material.

Second, as can be seen in Table 3, for the variables measured at both t2 and t3, strict measurement invariance can be shown for organizational identification and for social integration (equivalence of model form, factor loadings, item intercepts, and items’ residual variances; Putnick and Bornstein, 2016). In sum, the final structural equation model M4 had an acceptable model fit with *CFI* > 0.90, *RMSEA* < 0.05, and *SRMR* < 0.10 (Cheung and Rensvold, 2002; Schermelleh-Engel et al., 2003).

**Table 3:** Tests of measurement invariance.

Model	M1: Configural invariance	M2: Metric invariance	M3: Scalar invariance	M4: Residual invariance
*χ2* (*df*)	576.707 (380)	586.890 (386)	596.241 (392)	607.164 (400)
*CFI*	0.928	0.927	0.925	0.924
*RMSEA* [90% CI]	0.046 [0.038, 0.053]	0.046 [0.038, 0.053]	0.046 [0.038, 0.053]	0.046 [0.038, 0.053]
*SRMR*	0.079	0.083	0.083	0.083
Model comparison		M2-M1	M3-M2	M4-M3
Δ*χ^2^* (Δ*df*) for MLR		10.13 (6) n.s.	9.36 (6) n.s.	11.38 (8) n.s.
*AIC*	11750.387	11749.218	11746.389	11749.009
*BIC*	12155.355	12133.057	12109.099	12083.548
Sample-size adjusted *BIC*	11790.796	11787.517	11782.581	11782.390

CFI, comparative fit index; RMSEA, root-mean-square error of approximation; CI, confidence interval; SRMR, standardized root mean squared residual; AIC, akaike information criterion; BIC, bayesian information criterion; and n.s. non significant.

To examine the long-term effects of organizational support measures, we additionally modeled the indirect effects of support by the trainer, support by colleagues, and formal socialization on organizational identification (t3). For this analysis, we included direct paths from all three constructs to organizational identification (t3).

## 5. Results

As can be seen from the descriptive statistics in Table 4, the trainees rated all constructs of interest at a rather high level and dropout intention at a rather low level.

**Table 4:** Descriptive statistics.

Construct (timepoint)	*n*	*M*	*SD*
Social support by trainer (t1)	240	4.31	0.62
Social support by colleagues (t1)	228	4.25	0.68
Formal socialization (t2)	239	4.16	0.95
Social integration (t2)	241	4.38	0.75
Social integration (t3)	126	4.35	0.71
Organizational identification (t2)	241	3.44	0.89
Organizational identification (t3)	126	3.32	0.95
Self-perceived competence (t3)	126	4.01	0.66
Emotional engagement (t3)	126	3.84	0.96
Dropout intention (t3)	126	1.71	0.94

Accordingly, at the beginning of their training, the trainees experienced support from both the trainer and their colleagues. After 3 months, they reported a high level of formal introduction to their work/role as trainees, social integration, and organizational identification. After 9 months, the organizational identification of trainees was reduced slightly from t2 to t3, but the decrease was not significant [*t*(117) = 1.282; *p* > 0.05; *n* = 118]. The same applies to the minimal decrease in social integration from t2 to t3 [*t*(117) = 0.940; *p* > 0.05; *n* = 118]. Furthermore, the trainees experienced themselves as competent and emotionally engaged and had a low level of dropout intention.

**Figure 3:**
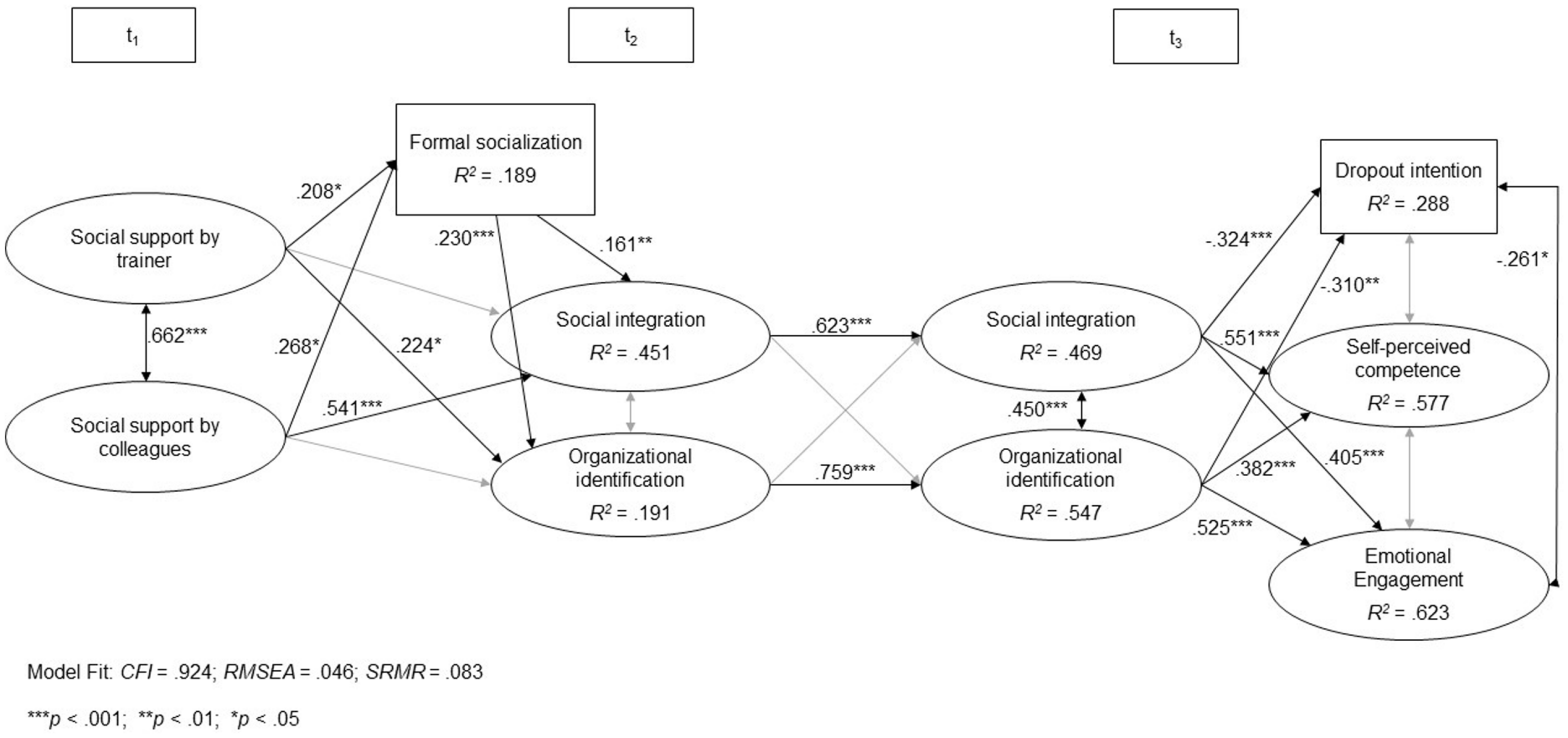
Relationships between different predictors and outcomes of organizational identification and social integration. Nonsignificant path in gray. To increase readability, we refrained from including the measurement model. The full model can be found in the Supplementary Material (Table A2).

Figure 3 shows the results of the structural equation model.

In line with the descriptive results, the autoregressive paths (research question 1) in the structural equation model (see Figure 3) showed a high level of stability of both organizational identification (*β* = 0.759; *p* < 0.001) and social integration (*β* = 0.623; *p* < 0.001) from t2 to t3.

The results also indicate that trainees’ organizational identification after 3 months of training (t2) was significantly positively affected by the social support by the trainer in the first weeks (*β* = 0.224; *p* < 0.05), as well as by formal socialization tactics implemented by the training company (*β* = 0.230; *p* < 0.001) but not by the social support by colleagues (research question 2). In contrast, social integration was only influenced by the social support by colleagues (*β* = 0.541; *p* < 0.001), not by the trainer. Furthermore, there was a significant effect of formal socialization on social integration (*β* = 0.161; *p* < 0.01).

The analysis of indirect effects (see Table 5) has shown that both support by the trainer and formal socialization tactics by the company at the beginning of the VET program indirectly and positively affected organizational identification at t3 *via* organizational identification at t2. The respective direct effects were insignificant. Regarding support by colleagues, neither the direct nor indirect effect on organizational identification (t3) was significant. Overall, the model depicted in Figure 3 was able to explain 19.1% of the variance in trainees’ organizational identification after 3 months and 54.7% of variance in organizational identification after 9 months of training.

**Table 5:** Indirect effects on organizational identification (t3).

Predictor	Mediator	Specific indirect			Total indirect		
		Std. Coef.	*p*	95% CI	Std. Coef.	*p*	95% CI
Support by trainer (t1)	Organizational identification (t2)	0.145	0.047	[0.025, 0.266]	0.175	0.031	[0.042, 0.308]
Formal socialization (t2)	Organizational identification (t2)	0.153	0.005	[0.064, 0.242]	0.120	0.034	[0.027, 0.213]

Outcome variable: organizational identification (t3). Std. Coef., standardized path coefficient; CI, confidence interval. Only significant paths are reported.

Regarding research question 3, the results have shown that self-perceived competence (*β* = 0.382; *p* < 0.001) and emotional engagement (*β* = 0.525; *p* < 0.001) at t3 were affected by organizational identification at t3 and by social integration at t3 (self-perceived competence: *β* = 0.511; *p* < 0.001; emotional engagement: *β* = 0.405; *p* < 0.001). Finally, trainees’ dropout intention at t3 was significantly reduced by organizational identification at t3 (*β* = −0.310; *p* < 0.001) and by social integration at t3 (*β* = −0.324; *p* < 0.001).

Concerning cross-lagged effects (research question 4), neither the path from organizational identification at t2 to social integration at t3 nor the path from social integration at t2 to organizational identification at t3 showed significant effects. This might be partly explained by the high stability of both constructs (see above). The only change between these two constructs was visible in the significant correlation between organizational identification and social integration at t3 (*r* = 0.45; *p* < 0.001), which was nonsignificant at t2.

## 6. Discussion

The present study of the trainees’ identification processes is particularly important in the context of VET because the transition from school to VET is a crucial step for young people and plays a significant role in determining their future career success. The formation of a vocational identity is an indicator of professional competence, and the organizational identification of employees is associated with many positive effects for individuals, companies, and society (Savickas, 1999; Rauner, 2007; Baethge et al., 2009; Seeber et al., 2019; Heinrichs et al., 2022). Nevertheless, there is a need for empirical longitudinal studies on the identification development of trainees in VET, as well as for findings on the predictors and effects of organizational identification (Heinrichs et al., 2022). So far, the state of research has been characterized by several single studies, and it is not (yet) possible to speak of a robust body of knowledge.

Against this background, our study has revealed on a descriptive level that trainees experience both a high level of support from trainers and colleagues at the beginning of their training and a high level of formal familiarization with their tasks during the probationary period. This goes hand in hand with a high degree of social integration, identification with the organization, self-perceived competence, and emotional engagement, as well as a comparatively low intention to drop out in the first 9 months of training. The finding that organizational identification is already rather high at an early stage of training is congruent with findings from other studies, even though these often focus on a later stage of training (e.g., Metzlaff, 2015; Forster-Heinzer, 2020; Böhn and Deutscher, 2021; Kirchknopf and Kögler, 2022). Concerning the development of trainees’ organizational identification (research question 1), there was a high stability of organizational identification from t2 to t3. On the one hand, this contradicts our first hypothesis and the findings of Heinemann et al. (2009) and Kirchknopf and Kögler (2022) of a significant decrease over the course of the first year of training. On the other hand, the findings are in line with the results of Fischer (2013) for the domain of health care and nursing, of Eisenbeiss and Otten (2008) for training groups of flight attendants, and of Schaubroeck et al. (2013), who state a correlation of *r* = 0.61 for organizational identification at the beginning and at the end of a 14-week residential entry program for U.S. Army soldiers. Both Heinemann et al. (2009) and Kirchknopf and Kögler (2022) have shown different developments in different training occupations. However, a distinction among the different occupations was not possible in our analysis because of sample size restrictions. Consequently, the differences between trainees from different training occupations might have been canceled out. Another explanation could be that changes in organizational identification only become apparent over a longer period of time so they cannot (yet) be depicted here because of the shorter observation period (also in Schaubroeck et al., 2013) compared with other studies that examined the development over the entire training period (Heinemann et al., 2009; Kirchknopf and Kögler, 2022). This can be tested by future analyses of currently ongoing data collections in our research project. In addition, again because of sample size restrictions, trainees’ personal characteristics could not be included in the analyses, such as their motivation to pursue a particular occupation or whether they currently attend a training program in the desired occupation or organization, which can have a positive impact on the organizational identification (Heinemann et al., 2009; Metzlaff, 2015) and its longer-term development (Eisenbeiss and Otten, 2008). Accordingly, further research is necessary to examine how the development of identification is affected by occupations and the corresponding differences in the training conditions or by underlying differences between trainees over the entire duration of the training.

Moreover, our findings highlight the positive effects of social support received by the trainer (hypothesis 2) at the beginning of the training period and by the formal socialization tactics employed by the company (hypothesis 4) for the development of organizational identification at t2 (research question 2). Furthermore, both support by the trainer at t1 and formal socialization tactics by the company at t2 have a longer-term impact on organizational identification; they indirectly and positively affect organizational identification after 9 months of training (t3) *via* organizational identification after 3 months of training (t2). Hence, our results support prior findings on the importance of the trainer (van Knippenberg et al., 2007; Metzlaff, 2015; Forster-Heinzer, 2020) and of formal onboarding measures employed by the company (Narayansany and Mat Isa, 2021) for the initial phase of VET. Interestingly, though, support by colleagues did not affect trainees’ organizational identification at t2 (contradicting hypothesis 3 and prior findings by Böhn and Deutscher, 2021 and Heinemann et al., 2009). One possible explanation could be that the trainees perceive their trainers primarily as responsible for the technical and professional aspects and see them as role models (Chan, 2014) and representatives of the organization, whereas the contact with colleagues involves more social aspects and is thus aimed at social integration. These considerations are supported by findings of Collin et al. (2008) who report the following findings for Finnish vocational students: “intentional and active actions, such as availability of individual guidance which students reported getting in the workplaces, or students’ active participation in the practices of the work community, seemed to promote their learning and vocational identity formation at work. Instead, social and interactive support, which students reported receiving, did not show a positive relation to students’ learning and vocational identity formation during their workplace learning periods” (p. 202). Another reason could lie in the fact that the majority of trainees in our sample completes the training in a large company with more than 250 employees. It is possible that at the time of the survey, right at the start of the training, the trainees had not yet received much specialist support from their colleagues and rather interact with the trainer due to the way the training was organized. Overall, our results suggest that trainers were more important for beginning trainees than their colleagues when regarding both short- and long-term effects on the trainees’ organizational identification. Nevertheless, collegial support was important because it was found to promote the social integration of the trainees.

With regard to effects (research question 3), organizational identification was found to be a positive predictor of trainees’ self-perceived competence (hypothesis 5) and emotional engagement (hypothesis 6). These results are in line with various findings on the relationship between identification and performance (Klotz et al., 2014; Kim et al., 2015; Böhn and Deutscher, 2021; Thole, 2021; Kirchknopf and Kögler, 2022) as well as between organizational identification and emotional engagement (e.g., Fischer, 2013; Karanika-Murray et al., 2015). Hence, our findings underline the importance of organizational identification as a central goal of VET programs and as a prerequisite of trainees’ engagement during the VET program and their ability to fulfill their work tasks (Lee, 2004; Heinemann et al., 2009; Heinrichs et al., 2022). Additionally, the negative effect of organizational identification at t3 on dropout intention at t3 affirms that organizational identification can serve as a protective factor for the intention to drop out of training, which confirms hypothesis 7 and is in line with other studies (Narayansany and Mat Isa, 2021; Kirchknopf and Kögler, 2022). The extent to which this affects the further course of training and possible completion of training must remain open at this point in time and can only be examined based on the data from later surveys that will be carried out in our research project. However, long-term positive effects can be expected based on the findings of Narayansany and Mat Isa (2021) and on the evidence showing an indirect and positive effect of employees’ organizational identification on job satisfaction *via* work engagement (Karanika-Murray et al., 2015). This seems desirable when it comes to the individual being able to successfully shape their career and life path, but also regarding their well-being and health. Moreover, this effect can benefit training companies regarding securing the demand for skilled workers, working climate, and customer satisfaction (e.g., Savickas, 1999; Metzlaff, 2015; Seeber et al., 2019; Thole, 2021).

Concerning the relationship between organizational identification and social integration (research question 4), only the correlation between these two constructs at t3 was significant. Neither the cross-lagged effects nor the correlation at t2 were significant. Kirchknopf and Kögler (2022) found that social integration can prevent a decline in organizational identification. Hence, our results could be an indicator that the relationship between social integration and organizational identification manifests at a later time during the VET program instead of at the very beginning. At the start of VET, the results indicate that organizational identification seemed to depend on factors other than social integration (e.g., the relationship with the trainer), but this changed over time. This assumption is supported by the findings of Metzlaff (2015) on trainees at the end of the first and end of the second year of apprenticeship, where the correlations of different facets of organizational identification with social integration are slightly higher than those with relationship with the trainer. Nevertheless, the evidence on the relationship and interaction between social integration and organizational identification is thin, which is why future research should further investigate this relationship. Although we found insignificant cross-lagged effects in our model, organizational identification and social integration proved to be very similar regarding development, predictors, and effects. This not only supports the assumption that organizational identification and social integration are positively related but is also in line with prior research (see Section 2.4). The only difference occurred regarding the influence of support: while support by the trainer was crucial for organizational identification, colleagues played an important role in social integration and, thus, can be seen as a valuable resource for socialization processes and professional and personal development during training.

The present study is subject to certain limitations that need to be taken into account. First, the surveyed trainees come from an occasional sample from a wide range of occupations and from different regions in Germany, with correspondingly different structural conditions for VET. Second, the start of data collection occurred in a difficult year for companies, specifically in the wake of the COVID-19 pandemic, which significantly limited their willingness to participate because of other challenges which resulted in a limited sample size. Therefore, we were not able to additionally examine the differences between training companies and training occupations. It would be highly plausible that training conditions and a company’s experiences with training programs (e.g., number of trainees, company size, and integration of trainees in existing work processes) and experiences of trainers in the training of trainees and their leadership would also affect trainees’ identification processes (e.g., Epitropaki and Martin, 2005; Klotz et al., 2014; Metzlaff, 2015; Forster-Heinzer, 2020; Ko and Choi, 2021). In addition, we were unable to conduct analyses differentiated by the personal characteristics of the trainees, for example, trainees’ professional motivation, desired occupation, or personality (e.g., Eisenbeiss and Otten, 2008; Metzlaff, 2015). Consequently, further research should examine the training conditions, characteristics of the training companies, and trainees’ personal characteristics more thoroughly to determine their role in the process of organizational identification. Third, we used only self-reported performance measures. For future research, it would be interesting to include competency scores based on established test instruments. This would, however, entail the challenge and task of having to develop competency tests not only for numerous training occupations (currently 324 training occupations; Bundesinstitut für Berufsbildung (BIBB), 2021), but also for different training years per occupation. This would far exceed the possibilities of an individual research project. Similarly, the realized dropouts of trainees may provide different information than self-reported dropout intentions, so both measurements should be combined. This was not possible in the present study because of the very low number of reported premature contract terminations. The fact that the current study is part of an ongoing research project with data collection not yet completed is the fourth limitation, in which, so far only the first 9 months of training—thus not the entire duration—can be considered. Finally, the operationalization of organizational identification is worth mentioning. Although we used an established scale (Mael and Ashforth, 1992), this did not allow for a differentiation of different dimensions, such as the cognitive, affective, behavioral, and evaluative dimensions of organizational identification (Metzlaff, 2015; Kirchknopf, 2020; Kirchknopf and Kögler, 2022). A more comprehensive assessment of organizational identification was not possible for test-economic reasons.

Despite these limitations, the results of our study are important for the organization of VET programs and can inform practitioners and companies aiming to support trainees’ socialization processes during their VET program and development as skilled employees of the company. First, the findings further highlight the importance of organizational identification for competences, engagement, and persistence intention of trainees in different occupations. This adds to prior research, which has often focused on selected occupations (e.g., Fischer, 2013; Metzlaff, 2015). Consequently, companies should aim to foster trainees’ organizational identification at the start of a VET program. Our results further suggest that achieving a high level of organizational identification at the beginning of a VET program can pay off because organizational identification is stable over the course of the first year of VET. When it comes to measures the companies can enact, support by trainers and formal socialization tactics produce both short- and long-term effects on organizational identification. Hence, companies should train their trainers in how to effectively support trainees in the initial phase of VET. Such training of trainers’ skills would not only pay off regarding trainees, but can also have an effect on employees who have been working in the company for a longer period of time. Here, Ko and Choi (2021) were able to show that leadership behaviors promoting positive work experiences, competencies, and performance among employees have a positive impact on employees’ organizational identification. Similarly, the findings from Epitropaki and Martin (2005) indicate that the positive effect of transformational leadership on organizational identification is significantly greater than the positive effect of transactional leadership. In addition, investing time and monetary resources in the implementation of specific onboarding measures designed to support trainees’ start in the training company seems worthwhile.

## Data availability statement

The raw data supporting the conclusions of this article will be made available by the authors, without undue reservation.

## Ethics statement

Ethical review and approval was not required for the study on human participants in accordance with the local legislation and institutional requirements. Written informed consent from the participants’ legal guardian/next of kin was not required to participate in this study in accordance with the national legislation and the institutional requirements.

## Author contributions

EM, SF, and SS contributed to conception and design of the study. EM organized the data set. EM and SF performed the statistical analysis and wrote the first draft of the manuscript. SS acquired the project funding and continued working on the first draft of the manuscript. All authors contributed to the article and approved the submitted version.

## Funding

This work was funded by the Deutsche Forschungsgemeinschaft (DFG—German Research Foundation) under Germany‘s Excellence Strategy—EXC-2035/1–390681379. Funding of the open access publication fees by the Publication Fund of the University of Konstanz.

## Conflict of interest

The authors declare that the research was conducted in the absence of any commercial or financial relationships that could be construed as a potential conflict of interest.

## Publisher’s note

All claims expressed in this article are solely those of the authors and do not necessarily represent those of their affiliated organizations, or those of the publisher, the editors and the reviewers. Any product that may be evaluated in this article, or claim that may be made by its manufacturer, is not guaranteed or endorsed by the publisher.

## Footnotes

^1^Organizational identification and organizational commitment are often used synonymously. Although the two constructs are related (organizational commitment is defined as an “individual’s attachment to the organization and her or his identification with the organization”; Nägele and Neuenschwander, 2014, 386), the two constructs have been shown to be empirically distinguishable (e.g., Mael and Tetrick, 1992). Conceptually, commitment refers to affective aspects while identification additionally refers to a cognitive, self-definitional component (see, e.g., Ashforth and Mael, 1989; van Knippenberg, 2000).

^2^ https://www.integrationatwork.de/

^3^Data on beginning trainees in VET in Germany is available from Bundesinstitut für Berufsbildung (BIBB) (2022) for 2020 or 2021 depending on the variable of interest. Our sample is comparable to the population of beginning trainees in dual VET programs in Germany regarding age (mean age in 2020: 19.9 years; proportion of trainees over 40 years old: 0.4%, just like 0.4% in our sample) and gender (proportion of female trainees in 2021: 49.4%). Regarding school-leaving certificates, however, our sample contains a higher share of trainees with higher education entrance qualification (only 23% in the population) and a lower share of lower secondary school-leaving certificates (23% in the population) than the population of trainees who started VET in 2020. Concerning migration background the information in the official data (Bundesinstitut für Berufsbildung (BIBB), 2022) are not as differentiated and offer only information about the citizenship of trainees (12% without German citizenship compared to 22% in our sample). These differences need to be taken into account when interpreting our results.
