# Supplementary material for: Development, predictors, and effects of trainees’ organizational identification during their first year of vocational education and training
Source: Front Psychol. 2023 Apr 17;14:1148251. doi: 10.3389/fpsyg.2023.1148251 (PMC10149868; doi:10.3389/fpsyg.2023.1148251)
Supplement: Supplementary file 1 [file Table_1.docx]

**Table A1**

*Drop-out analyses: Comparison of participants included in the analyses and participants excluded in the analyses*

| Variable | Range | Included | | | Excluded | | | Difference | | |
| --- | --- | --- | --- | --- | --- | --- | --- | --- | --- | --- |
|  |  | *n* | *M* | *SD* | *n* | *M* | *SD* | *t* | *p* | *\|d\|* |
| Gender (t0) | 0 = woman  1 = man | 250 | 0.56 | 0.50 | 232 | 0.56 | 0.50 | -0.08 | .94 | 0.01 |
| Age (t0) | years | 249 | 21.19 | 4.48 | 231 | 21.36 | 5.33 | 0.37 | .71 | 0.03 |
| Born in Germany (t0) | 0 = no  1 = yes | 250 | 0.75 | 0.43 | 232 | 0.75 | 0.43 | -0.05 | .96 | 0.01 |
| Second generation migrant (t0) | 0 = no  1 = yes | 250 | 0.21 | 0.41 | 232 | 0.27 | 0.45 | 1.63 | .10 | 0.15 |
| German as mother tongue (t0) | 0 = no  1 = yes | 247 | 0.67 | 0.47 | 229 | 0.62 | 0.49 | -1.19 | .24 | 0.11 |
| Highest education (t0) | 1 = No diploma  4 = University Degree | 246 | 3.20 | 0.92 | 231 | 3.06 | 0.97 | -1.60 | .11 | 0.15 |
| Support by trainer (t1) | 1 = not true  5 = true | 240 | 4.31 | 0.62 | 150 | 4.22 | 0.73 | -1.28 | .20 | 0.14 |
| Support by colleagues (t1) | 1 = not true  5 = true | 228 | 4.25 | 0.68 | 132 | 4.20 | 0.73 | -0.60 | .55 | 0.07 |

*Note*. *p* = two-tailed *p*-value; *d* = effect size Cohen’s *d*; t0 = presurvey; t1 = week 1-12 (see Figure 2).
