# Supplementary material for: Development, predictors, and effects of trainees’ organizational identification during their first year of vocational education and training
Source: Front Psychol. 2023 Apr 17;14:1148251. doi: 10.3389/fpsyg.2023.1148251 (PMC10149868; doi:10.3389/fpsyg.2023.1148251)
Supplement: Supplementary file 2 [file Table_2.docx]

**Table A2**

*Relationships between different predictors and outcomes of organizational identification and social integration (full model)*

|  | *β* | *SE* | *p* |
| --- | --- | --- | --- |
| *Measurement model* |  |  |  |
| Social support by trainer (t1) |  |  |  |
| Item 1 | .758 | .053 | .000 |
| Item 2 | .881 | .035 | .000 |
| Item 3 | .795 | .041 | .000 |
| Social support by colleagues (t1) |  |  |  |
| Item 1 | .817 | .040 | .000 |
| Item 2 | .860 | .034 | .000 |
| Item 3 | .771 | .026 | .000 |
| Social integration (t2) |  |  |  |
| Item 1 | .780 | .041 | .000 |
| Item 2 | .848 | .027 | .000 |
| Item 3 | .841 | .029 | .000 |
| Item 4 | .846 | .029 | .000 |
| Social integration (t3) |  |  |  |
| Item 1 | .758 | .043 | .000 |
| Item 2 | .831 | .028 | .000 |
| Item 3 | .823 | .029 | .000 |
| Item 4 | .828 | .029 | .000 |
| Organizational identification (t2) |  |  |  |
| Item 1 | .508 | .049 | .000 |
| Item 2 | .593 | .049 | .000 |
| Item 3 | .825 | .028 | .000 |
| Item 4 | .796 | .032 | .000 |
| Organizational identification (t3) |  |  |  |
| Item 1 | .530 | .051 | .000 |
| Item 2 | .615 | .052 | .000 |
| Item 3 | .840 | .027 | .000 |
| Item 4 | .812 | .031 | .000 |
| Self-perceived competence (t3) |  |  |  |
| Item 1 | .738 | .056 | .000 |
| Item 2 | .737 | .081 | .000 |
| Item 3 | .388 | .094 | .000 |
| Emotional engagement (t3) |  |  |  |
| Item 1 | .864 | .044 | .000 |
| Item 2 | .815 | .034 | .000 |
| Item 3 | .926 | .026 | .000 |
| *Path model* |  |  |  |
| Organizational identification (t2) on |  |  |  |
| Social support by trainer (t1) | .224 | .105 | .033 |
| Social support by colleagues (t1) | .085 | .115 | .458 |
| Formal socialization (t2) | .230 | .072 | .001 |
| Social integration (t2) on |  |  |  |
| Social support by trainer (t1) | .068 | .113 | .548 |
| Social support by colleagues (t1) | .541 | .112 | .000 |
| Formal socialization (t2) | .161 | .058 | .006 |
| Formal socialization (t2) on |  |  |  |
| Social support by trainer (t1) | .208 | .099 | .036 |
| Social support by colleagues (t1) | .268 | .110 | .015 |
| Organizational identification (t3) on |  |  |  |
| Organizational identification (t2) | .759 | .081 | .000 |
| Social integration (t2) | -.073 | .080 | .362 |
| Social integration (t3) on |  |  |  |
| Organizational identification (t2) | .150 | .089 | .092 |
| Social integration (t2) | .623 | .076 | .000 |
| Self-perceived competence (t3) on |  |  |  |
| Organizational identification (t3) | .382 | .113 | .001 |
| Social integration (t3) | .511 | .097 | .000 |
| Emotional engagement (t3) on |  |  |  |
| Organizational identification (t3) | .525 | .084 | .000 |
| Social integration (t3) | .405 | .084 | .000 |
| Dropout intention (t3) on |  |  |  |
| Organizational identification (t3) | -.310 | .098 | .002 |
| Social integration (t3) | -.324 | .090 | .000 |
| Organizational identification (t2) with Social integration (t2) | .081 | .091 | .374 |
| Organizational identification (t3) with Social integration (t3) | .450 | .121 | .000 |
| Dropout intention (t3) with |  |  |  |
| Self-perceived competence (t3) | -.055 | .132 | .678 |
| Emotional engagement (t3) | -.261 | .126 | .039 |
| Self-perceived competence (t3) with Emotional engagement (t3) | .110 | .176 | .533 |
| Social support by trainer (t1) with Social support by colleagues (t1) | .662 | .060 | .000 |
| Organizational identification |  |  |  |
| Item 1 (t2) with Item 1 (t3) | .460 | .083 | .000 |
| Item 2 (t2) with Item 2 (t3) | .518 | .077 | .000 |
| Item 3 (t2) with Item 3 (t3) | .395 | .091 | .000 |

*Note*. on = regressed on; with = correlation.
